# *Betula etnensis* Raf. (Betulaceae) Extract Induced HO-1 Expression and Ferroptosis Cell Death in Human Colon Cancer Cells

**DOI:** 10.3390/ijms20112723

**Published:** 2019-06-03

**Authors:** Giuseppe Antonio Malfa, Barbara Tomasello, Rosaria Acquaviva, Carlo Genovese, Alfonsina La Mantia, Francesco Paolo Cammarata, Monica Ragusa, Marcella Renis, Claudia Di Giacomo

**Affiliations:** 1 Department of Drug Science, Section of Biochemistry, University of Catania, Viale A. Doria 6, 95125 Catania, Italy; g.malfa@unict.it (G.A.M.); btomase@unict.it (B.T.); alfy.lamantia@gmail.com (A.L.M.); renis@unict.it (M.R.); cdigiaco@unict.it (C.D.G.); 2 Department of Biomedical and Biotechnological Sciences, Microbiology Section University of Catania, 95125 Catania, Italy; gnv.carlo@gmail.com; 3 Institute of Bioimaging and Molecular Physiology, National Council of Research (IBFM-CNR), 90015 Cefalù (PA), Italy; fracammarata@gmail.com; 4 National Institute of Nuclear Physics, South National Laboratory (LNS-INFN), 95125 Catania, Italy; 5 Department of Experimental and Clinical Medicine, University Magna Graecia of Catanzaro, 88100 Catanzaro, Italy; m.ragusa@unicz.it

**Keywords:** Colon cancer, *Betula etnensis* Raf., oxidative stress, heme oxigenase-1, ferroptosis, thiol groups

## Abstract

*Betula etnensis* Raf. (Birch Etna) belonging to the Betulaceae family grows on the eastern slope of Etna. Many bioactive compounds present in Betula species are considered promising anticancer agents. In this study, we evaluated the effects of *B. etnensis* Raf. bark methanolic extract on a human colon cancer cell line (CaCo2). In order to elucidate the mechanisms of action of the extract, cellular redox status, cell cycle, and heme oxygenase-1 (HO-1) expression in ferroptosis induction were evaluated. Cell viability and proliferation were tested by tetrazolium (MTT) assayand cell cycle analysis, while cell death was evaluated by annexin V test and lactic dehydrogenase (LDH) release. Cellular redox status was assessed by measuring thiol groups (RSH) content, reactive oxygen species (ROS) production, lipid hydroperoxide (LOOH) levels and (γ-glutamylcysteine synthetase) γ-GCS and HO-1 expressions. The extract significantly reduced cell viability of CaCo2, inducing necrotic cell death in a concentration-depending manner. In addition, an increase in ROS levels and a decrease of RSH content without modulation in γ-GCS expression were detected, with an augmentation in LOOH levels and drastic increase in HO-1 expression. These results suggest that the *B. etnensis* Raf. extract promotes an oxidative cellular microenvironment resulting in CaCo2 cell death by ferroptosis mediated by HO-1 hyper-expression.

## 1. Introduction

After prostate cancer in men, breast cancer in women and lung cancer, the colorectal cancer (CRC) represents the second leading cause of death in the Western world in both males and females. The association between nutrition and colon cancer has extensively been investigating by many studies but is controversial because of the diet shows a causal and protective role in the CRC development [1,2].

It is well known that the consumption of vegetables is correlated with a low incidence of cancer and, in particular, is effective in the prevention and reduction of CRC risk [3,4]. A plausible reason might be plant foodstuff is a good source of fibers, folate vitamins, various antioxidants, and other bioactive compounds including polyphenols, terpenoids, saponins, and carotenoids [5,6,7]. Besides their well-known anti-oxidant activities, they have been reported to be anti-mutagenic and/or anti-carcinogenic and to possess several other biological activities. In addition, natural antioxidants are known to have a dual face, behaving as pro-oxidant compounds after reacting directly with reactive oxygen species (ROS) in the presence of transition metal ions, such as copper and iron. This increase of cellular ROS to cytotoxic level may generate secondary oxidative damage and induce a selective killing of cancer cells by a variety of ways including ferroptosis, a non-apoptotic form of cell death characterized by the high expression of heme oxygenase-1 (HO-1) and accumulation of lipid hydroperoxides (LOOH) [8]. The role of heme oxygenase-1 (HO-1) in cancer biology is poorly understood. In fact, HO-1 has been described as survival molecule because of its anti-apoptotic and pro-angiogenic effects in several cancer types and its modulation can be induced by several natural compounds such as polyphenols and terpenoids [5].

Today potential usefulness of natural compounds as anti-cancer agents has to be ascribed prevalently to improve patient quality of life and to support conventional chemotherapy or radiotherapy. In the genus *Betula* (Betulaceae) there belongs common trees and shrubs of the boreal and north temperate zones. The bioactivities of Betula species are well documented, in fact the Betula was largely used in human and veterinary medicine to treat various diseases. Betula compounds have a wide variety of properties including anticancer, anti-inflammatory, and immunomodulatory beyond being antioxidant [9,10,11]. 

Currently, of particular interest are anticancer activities showed in vitro by some constituents isolated from these plants, which displayed cytotoxic effects on neuroblastoma, melanoma, medulloblastoma, and Ewing’s sarcoma cells [10,12]. In particular, it has been reported that betulinic acid may be considered a promising anticancer agent [10,13]. Commonly known as Birch Etna, *B. etnensis* Raf. is a medium-sized deciduous tree typically reaching 5–20 m tall, which belongs to the family Betulaceae. It grows on Mt. Etna volcano, at an altitude between 1200 and 2000 m and its ivory colored bark, in particular in the young branches, is rich of resinous chemicals secreted by the numerous glands present [14]. Polyphenols, terpenoids, betulin, betulinic acid, and ursolic acid are the main constituents present in the *B. etnensis* Raf. bark. As for as our knowledge no literature data are present on biological activities of *B. etnensis* Raf. 

In the present study, we evaluated, for the first time, the effects of *B. etnensis* Raf. bark methanolic extract on a human colon cancer cell line (CaCo2). In order to elucidate the mechanisms of action of this extract, cell viability, annnexin, lactic dehydrogenase (LDH) release, ROS production, thiol groups (RSH) content, lipid peroxidation (LOOH) levels, cell cycle, HO-1, and γ-GCS protein expression were evaluated.

## 2. Results

### 2.1. MTT Bioassay

**Figure 1:**
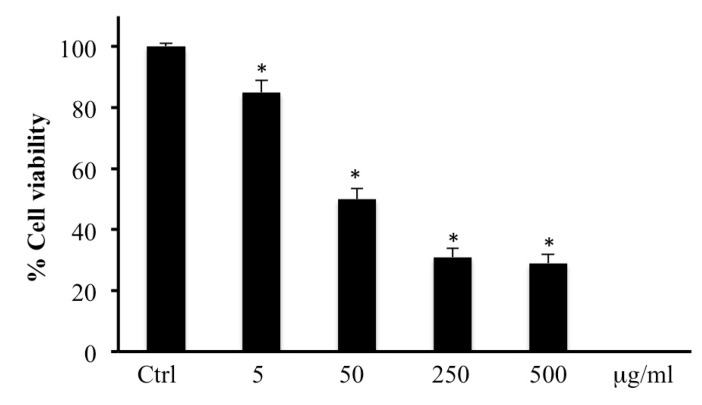
Cell viability in human colon cancer cell line (CaCo2) cells untreated and treated for 72 h with methanolic extract of *B. etnensis* Raf. at different concentrations (5–500 µg/mL). Values are the mean ± SD of four experiments in triplicate. * Significant vs. untreated control cells: *p* < 0.001.

Methanolic extract of bark from *B. etnensis* Raf. (5, 50, 250, or 500 µg/mL) was able to significantly reduce in a dose dependent manner cell viability (Figure 1); because 250 and 500 µg/mL of methanolic extract showed similar effects, in the other subsequent experiments we omitted 500 µg/mL of extract.

### 2.2. LDH Release 

**Figure 2:**
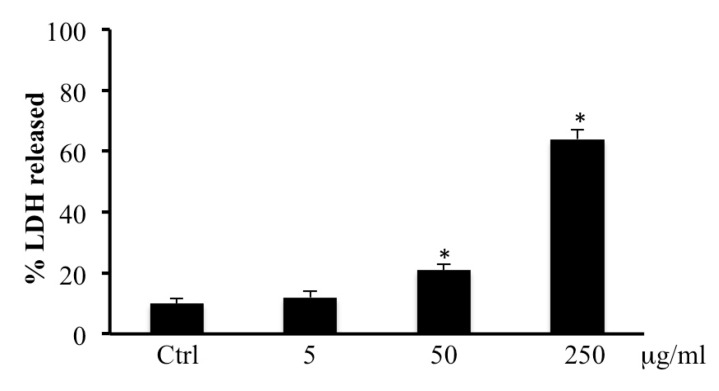
Lactic dehydrogenase (LDH) released in CaCo2 cells untreated and treated for 72 h with methanolic extract of *B. etnensis. Raf.* at different concentrations (5–250 µg/mL). Values are the mean ± SD of four experiments in triplicate. * Significant vs. untreated control cells: *p* < 0.001.

As shown in Figure 2, after 72 h of incubation with methanolic extract of bark from *B. etnensis* Raf. a statistically significant increase in LDH release was observed in CaCo2 cells treated with 50 and 250 µg/mL of extract.

### 2.3. Annexin V and Dead Cell Evaluation

A slight non-significant increase in the percentage of total apoptotic cells from 9.20 ± 1.25% to 11.75 ± 0.40% after treatment with 50–250 µg/mL of the extract was observed (Table 1). The annexin-V/7-AAD results confirm that most of antiproliferative activity of *B. etnensis* Raf. observed by viability assay is mediated by necrosis. 

### 2.4. ROS Levels

**Figure 3:**
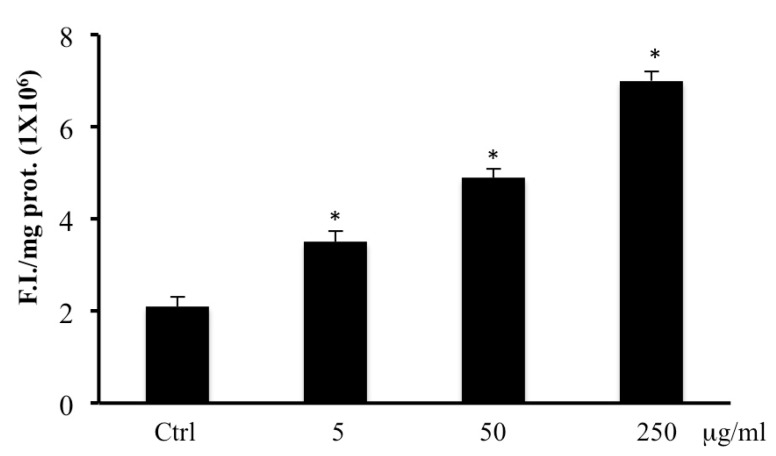
Intracellular oxidants in CaCo2 cells untreated and treated for 72 h with methanolic extract of *B. etnensis* Raf. at different concentrations (5–250 µg/mL). Values are the mean ± SD of four experiments in triplicate. * Significant vs. untreated control cells: *p* < 0.001.

Data reported in Figure 3 demonstrate that exposure of CaCo2 cells to several concentrations of *B. etnensis* Raf. methanolic extracts resulted in a significant increase in radical species, as revealed by fluorescence intensity. 

### 2.5. LOOH Levels

**Figure 4:**
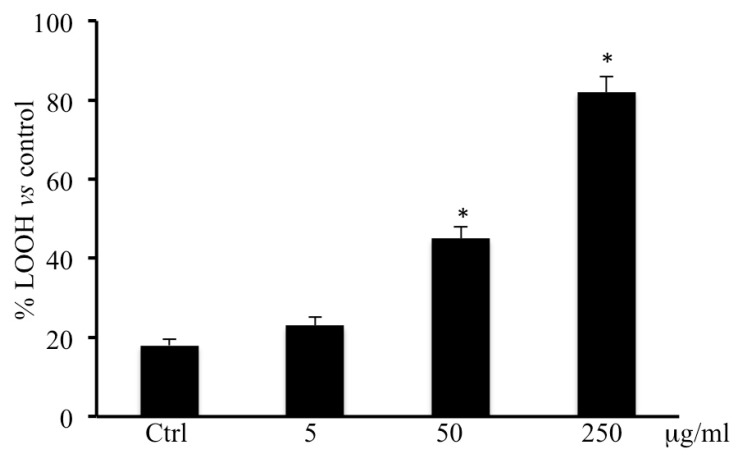
LOOH levels in CaCo2 cells untreated and treated for 72 h with methanolic extract of *B. etnensis* Raf. at different concentrations (5–250 µg/mL). Values are the mean ± SD of four experiments in triplicate. * Significant vs. untreated control cells: *p* < 0.001.

Figure 4 shows that the addition of methanolic extract of *B. etnensis* Raf. at 5, 50, and 250 μg/mL for 72 h induced a significant and dose-dependent increase in LOOH levels with respect to untreated CaCo2 cells. 

### 2.6. Total Thiol Groups

**Figure 5:**
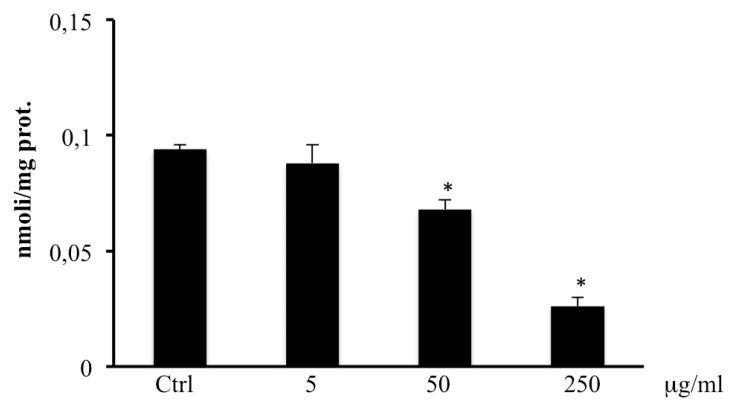
Thiol groups in CaCo2 cells untreated and treated for 72 h with methanolic extract of *B. etnensis* Raf. at different concentrations (5–250 µg/mL). Values are the mean ± SD of four experiments in triplicate. * Significant vs. untreated control cells: *p* < 0.001.

Treatment of cells with 50–250 µg/mL methanolic extract of *B. etnensis* Raf. resulted in a significant reduction in RSH levels (Figure 5). Instead, the lowest concentration of methanolic extract of *B. etnensis* Raf. did not alter the levels of RSH groups with respect to the control. 

### 2.7. γ-GCS Determination 

**Figure 6:**
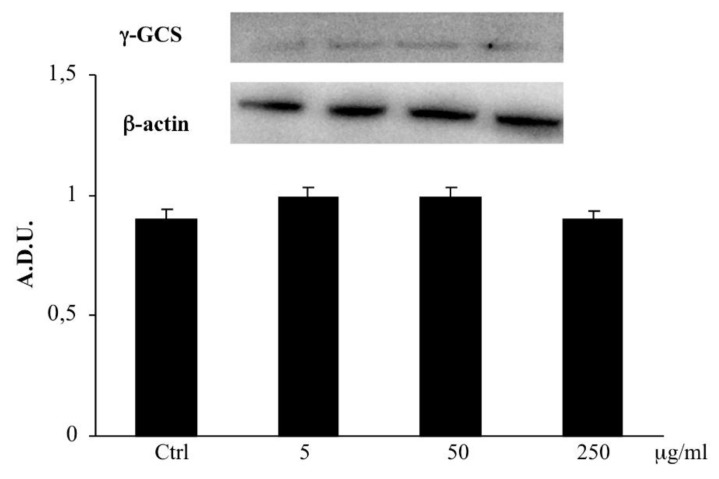
Immunoblotting of (γ-glutamylcysteine synthetase) γ-GCS levels in CaCo2 cells untreated and treated for 72 h with methanolic extract of *B. etnensis* Raf. at different concentrations (5–250 µg/mL). Values are the mean ± SD of four experiments performed in triplicate. * Significant vs. untreated control cells: *p* < 0.001.

No significant change in γ-GCS expression was observed in CaCo2 cells treated with the extract of *B. etnensis* Raf. with respect to untreated cells (Figure 6). 

### 2.8. HO-1 by ELISA

**Figure 7:**
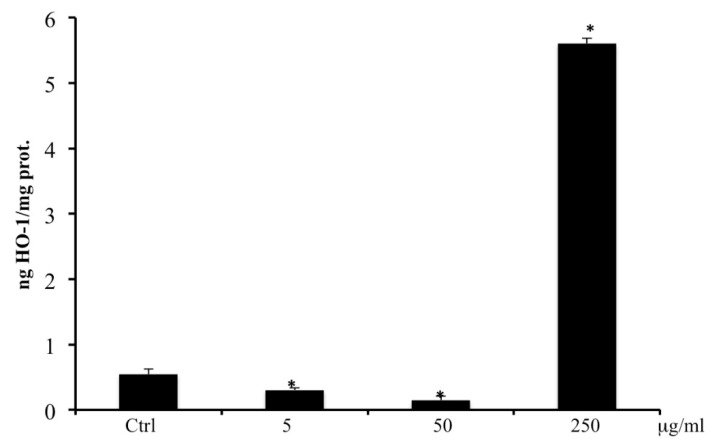
Heme oxygenase-1 (HO-1) levels in CaCo2 cells untreated and treated for 72 h with methanolic extract of *B. etnensis* Raf. at different concentrations (5–250 µg/mL). Values are the mean ± SD of four experiments in triplicate. * Significant vs. untreated control cells: *p* < 0.001.

Results reported in Figure 7 show that the addition of 250 μg/mL of extract to CaCo2 cells, caused a significant increase in HO-1 protein expression (Figure 7). 

### 2.9. Cell Cycle Analysis

**Figure 8:**
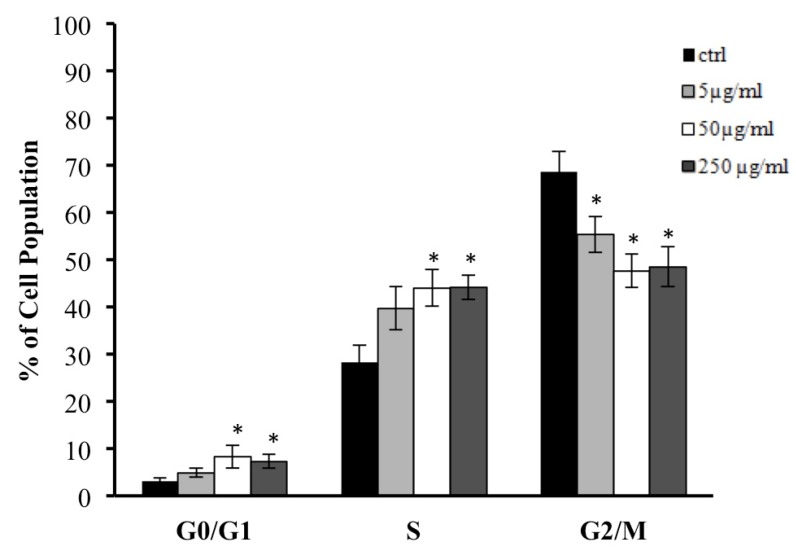
*B. etnensis* Raf. induces a cell cycle arrest at both G0/G1 and S phases in CaCo2 cells. The panel shows bar graph representing quantified values of the flow cytometry data. The graphs show the mean ± SD of four independent cell cycle experiments. (*) For values that are significantly different from the untreated control. * Significant vs. untreated control cells: *p* < 0.001.

When CaCo2 cells were treated with increasing concentrations of *B. etnensis* Raf. for 72 h, a dose dependent cell cycle arrest at G0/G1 and S phases were evident with concomitant marked decrease in the G2/M phase. After the treatment with 50 μg/mL of *B. etnensis* Raf., cell population in G0/G1 and S phases increased by ~5.14% and ~16% respectively, whereas cells in G2/M were decreased by ~21% (Figure 8). The same trend was observed with 250 μg/mL of extract. 

## 3. Discussion

Tumors of the digestive tract, particularly CRC, are among the most common forms of cancer, with thousands of deaths worldwide per year. Chemotherapy and radiation therapy are the main treatments with significant side effects. Among cancers, CRC is the most responsive to dietary modification, in fact, several studies demonstrated that approximatively 75% of all sporadic cases of CRC are directly influenced by diet and that dietary modification is a feasible strategy for reducing CRC risk [5,15,16]. The consumption of fruit and vegetables and herbs is associated with a low incidence of cancer [7,17] and this may be partly due to the presence of several bioactive natural compounds in plants [6]. 

Previous studies have shown that betulinic acid is cytotoxic for different types of human cancer cells [10,13,18]. In this study, we evaluated the anti-cancer activity of *B. etnensis* Raf. methanolic extract in CaCo2 cells.

In order to evaluate cytotoxic effects of the methanolic extract, we first conducted an MTT test. The results, showed in Figure 1, indicate that cell viability is significantly reduced by the extract. Cell viability reaches a plateau at 250 μg/mL with a 70% decrease and the same result was obtained with 500 μg/mL so the highest concentration of treatment used in next experiments was 250 μg/mL of *B. etnensis* Raf. methanolic extract.

In consideration of the results obtained on cell viability, we performed LDH assay in medium to evaluate cell membrane disruption which is accompanied by necrotic cells death. Results showed a dose-dependent necrotic effect with a maximum at 250 μg/mL in line with results obtained by MTT assay (Figure 2). These results were confirmed by annexin V levels, in fact, treatment with *B. etnensis* Raf. methanolic extract did not induce apoptotic cell death (Table 1). In order to verify the role of oxidative stress in the mechanism of cell death, the determination of ROS and LOOH was performed. Clearly, the treatments with *B. etnensis* Raf. extract induced an increase in ROS levels in CaCo2 tumor cell line, indicating that the extract does not act as an antioxidant agent, but rather exerts its cytotoxic effect as a pro-oxidant with concomitant increase lipid peroxidation particularly evident at 250 μg/mL (Figure 3 and Figure 4).

This result suggests that the extract may exert its activity by destabilizing the cellular redox balance so involving intracellular factors probable targets of ROS. Certainly the high production of ROS is a key factor that contributes to carcinogenesis. Within a neoplastic cell the persistent oxidative stress may be responsible for the activation of growth factor pathways and the increased resistance to apoptosis [19]. In fact, ROS have been shown to act as second messengers [20,21], stimulating the transduction of intracellular signals [22,23].

Recently, it has been suggested that GSH, as well as the main thiol responsible for maintaining the intracellular redox state, through thiol/disulfide exchange reactions, may be involved in the redox regulation of this type of signal [20,21,22,23,24]. 

In our study, the treatment of CaCo2 cells with the extract causes a dose-dependent reduction of intracellular RSH levels (Figure 5). To further confirm the involvement of the antioxidant defense systems in the mechanism of action of the extract, we also evaluated the expression of γ-GCS, a key enzyme in glutathione synthesis the expression of which in cancer cells is involved in tumor aggressiveness and chemo-resistance. The Western blot analysis of γ-GCS showed that the extract does not significantly influence the expression of the protein at all concentrations tested, suggesting that decreased RSH levels are most due to an inhibition of GSH synthesis and to a depletion for excessive ROS reactions (Figure 6). All these results confirms the persistence of a pro-oxidative imbalance induced by *B. etnensis* Raf. methanolic extract.

To further understand the mechanism of action of the extract, under the same experimental conditions, the expression of HO-1, one of the main cellular defense mechanisms against oxidative stress was evaluated. The role of HO-1 in tumors is still not very clear [25]. Results obtained in the present study show that the *B. etnensis* Raf. extract may exert a dual effect on HO-1 expression: at low concentration (5–50 μg/mL) it reduced HO-1 expression whereas, at higher concentrations (250 μg/mL) induced a significant increase in HO-1 expression (Figure 7). The reported inverse correlation between GSH levels and HO-1 expression is only confirmed at the highest concentration of the extract [26]. 

The HO-1 effect in cancer cells is not yet clear, but it is wildly documented that an HO-1 over-expression confers resistance to chemotherapy and radiation therapy. This protective effect could be due to its reaction products such as CO or biliverdin/bilirubin [27,28]. In spite of its implication in tumor initiation, angiogenesis, and metastasis, excessively increased expression of HO-1 in tumor cells may lead to cell death through a process called ferroptosis [29,30,31,32] In fact, a lot of evidence showed that HO-1 induces ferroptosis through an increase of ROS production mediated by iron accumulation [33,34,35] and accompanied by augmentation lipid peroxidation and glutathione depletion. Results obtained in the present study, at the highest concentration of the extract, may suggest a ferroptotic cell death.

The antiproliferative effect of extract was also demonstrated by the analysis of the cytometric flow, in fact the treatment induced cell cycle arrest at G0/G1 and S phases and a concomitant decrease in the G2/M phase.

Results obtained in the present research demonstrated that methanolic extract of *B. etnensis* Raf. by inducing ROS production and decreasing antioxidant cellular defense, elicited an imbalance in intracellular redox status. This effect, in turn, leads to a higher lipid peroxidation and a drastic rise in HO-1 activity, letting us to hypothesize the induction of ferroptosis, a non-programmed cell death.

## 4. Materials and Methods 

### 4.1. Chemicals

All solvents, chemicals and reference compounds were purchased from Sigma-Aldrich (Milano, Italy) except as otherwise specified.

### 4.2. Plant Collection and Preparation of Extract

*B. etnensis* Raf. bark was collected in the area around Linguaglossa (Catania, Italy) in November 2018. The specimen was obtained and authenticated by botanist R. Acquaviva, Department of Drug Science, Section of Biochemistry, University of Catania, Italy. A voucher specimen of the plant (No. 36/03) was deposited in the herbarium of the same Department. Dried bark from *B. etnensis* Raf. (50 g) was extracted at 80 °C in 70% methanol for 4 h. The extract was then filtered and evaporated to dryness under reduced pressure with a rotatory evaporator. The values of total phenolic and flavonoid content were 45 µM ± 0.05 gallic acid and 3.80 µM ± 0.09 catechin. 

### 4.3. Cell Culture and Treatments 

CaCo2 colon rectal cancer cells were obtained from ATCC cell bank (Rockville, MD, USA), were propagated in DMEM (Gibco BRL, Life Technologies, city, country) supplemented with 10% fetal calf serum, 1% sodium pyruvate, 1% L-glutamine solution, and 1% streptomycin/penicillin. 

After 24 h of incubation in a humidified atmosphere of 5% CO_2_ at 37 °C to allow cell attachment, the cells were treated for 72 h with different concentrations (5, 50, 100, 250 or 500 µg/mL) of the methanolic extract, previously dissolved in the minimum amount of dimethyl sulfoxide (DMSO) and diluted with medium.

### 4.4. MTT Bioassay

MTT assay was performed to assess cell viability on a 96 multiwell plate (8 × 103 cells/well). This assay measures the conversion of tetrazolium salt to yield colored formazan in the presence of metabolic activity. The amount of formazan is proportional to the number of living cells [36]. The optical density was measured with a microplate spectrophotometer reader (Titertek Multiskan, Flow Laboratories, Helsinki, Finland) at λ = 570 nm. Results are expressed as percentage cell viability respect to control (untreated cells).

### 4.5. Lactic Dehydrogenase Release

LDH release was measured to evaluate cell necrosis as a result of cell membrane disruption. LDH activity was measured spectrophotometrically in the culture medium and in the cellular lysates, at λ = 340 nm by analyzing NADH reduction [5]. The percentage of LDH release was calculated from the total sum of the enzymatic activity present in the cellular lysate and of that in the culture medium. Results are expressed as percentage in LDH released.

### 4.6. Annexin V and Dead Cell Evaluation 

The percentage of cells undergoing apoptosis after treatment with *B. etnensis* Raf. was evaluated by Muse™ Annexin V & Dead cell kit (Catalog No. MCH100105, Millipore, Milan, Italy) according to the manufacture’s protocol. Briefly for cells staining, 100 µL of the Muse™ Annexin V & Dead Cell Reagent was added to 100 µL of cell suspension, mixed thoroughly by vortexing and incubated for 20 min at room temperature in the dark. Then samples were analyzed by Muse™ Cell Analyzer (Millipore).

### 4.7. Reactive Oxygen Species Assay 

Dichlorofluorescein diacetate (DCFH-DA) assay was performed to quantify ROS levels as previously described [37]. The fluorescence intensity was detected by fluorescence spectrophotometry (excitation, λ = 488 nm; emission, λ = 525 nm). Results are expressed as fluorescence intensity/mg protein and, for each sample the total protein content was determined using the Sinergy HTBiotech instrument by measuring the absorbance difference at λ = 280 and λ = 260. Protein content was determined using the Sinergy HTBiotech instrument by measuring the absorbance difference at λ = 280 and λ = 260.

### 4.8. Determination of Lipid Hydroperoxide Levels 

LOOH levels were evaluated by oxidation of Fe^+2^ to Fe^+3^ that in the presence of xylenol orange forms a Fe^3+^-xylenol orange complex which can be measured spectrophotometrically at λ = 560 nm [38]. Results are expressed as percentage increase respect to control (untreated cells).

### 4.9. Total Thiol Group Determination 

RSH groups were measured by using a spectrophotometric assay as previously described [39]. Results are expressed in nmol/mg protein.

### 4.10. Western Blotting

CaCo2 cells were harvested using cell lysis buffer; cell lysates were collected for Western blot analysis and the level of γ-GCS expression was visualized by immunoblotting with primary antibody against γ-GCS as previously described [5]. The anti- γ-GCS was used in 1:1000 dilution in TBST solution containing 5% not fat milk and detected with secondary antibody conjugated to horseradish peroxidase and Pierce ECL Plus substrate solution (Thermo Scientific, Rockford, IL, USA). Beta-actin, was used as a loading control to normalize the expression level of γ-GCS protein. The result was expressed in arbitrary densitometric units (ADU).

### 4.11. HO-1 Protein Expression

HO-1 protein concentration in cellular lysates was measured using a commercial HO-1 ELISA kit, according to the manufacturers’ instructions. A standard curve generated with purified HO-1 was used to calculate HO-1 concentration from the absorbance at λ = 450 nm detected for each sample [40]. Detection limits were 0.78–25 ng/mL as reported by the manufacturer. Results are expressed as ng/mg protein.

### 4.12. Cell Cycle Analysis

Distribution of cell cycle was evaluated by flow cytometry using Muse™ Cell Cycle Kit (Catalog No. MCH100106, Millipore, Milan, Italy) according to the manufacture’s guidelines. Briefly, CaCo2 cells were treated with different concentration of *B. etnensis* Raf. (5, 50, 250 μg/mL) for 72 h. After incubation, the cells were harvest, washed with PBS and fixed with ice-cold 70% ethanol at −20 °C for 24 h. Then 200 μL of cell suspension (1 × 106 cells) was washed with PBS, stained with 200 μL of Muse™ Cell Cycle Reagent for 30 min in the dark at 37 °C and subsequently analyzed by Muse™ Cell Analyzer (Millipore).

### 4.13. Statistical Analysis

One-way analysis of variance (ANOVA) followed by Bonferroni’s *t*-test was performed in order to estimate significant differences among groups. Data were reported as mean values ± SD and differences among groups were considered to be significant at *p* < 0.005. 

## 5. Conclusions

These findings confirm the growing body of evidence on the bioactivities of *B. etnensis* Raf. and its potential impact on cancer therapy and human health. Our data demonstrate that exposure of CaCo2 cancer cells to *B. etnensis* Raf. extract decreased cell proliferation and induced necrotic death, suggesting that this extract may represent a subject for further studies on drug discovery, also helpful for cancer integrative medicine.

Moreover, our results show a prospective application of *B. etnensis* Raf. in the HO-1 mediated ferroptosis as a chemotherapeutic strategy against tumor. Further research should be carried out to better clarify the involvement of HO-1 in the mechanism of action of this promising anticancer medicinal plant.

## Figures and Tables

**Table 1:** Annexin V in CaCo2 cells untreated and treated for 72 h with methanolic extract of *B. etnensis* Raf. at different concentrations (5–250 µg/mL). Values are the mean ± SD of four experiments in triplicate. * Significant vs. untreated control cells: *p* < 0.001.

	Live	Early Apoptosis	Late Apoptosis	Dead Cells	Total Apoptosis
CTRL	96.0 ± 0.045%	0%	1.64 ± 0.09%	2.36 ± 0.06%	1.64 ± 0.05%
5 µg/mL	92.30 ± 0.055%	0%	2.1 ± 0.035%	4.9 ± 0.065%	2.1 ± 0.08%
50 µg/mL	84.70 ± 0.08% *	0%	9.20 ± 0.09%	6.10 ± 0.1%	9.20 ± 1.25%
250 µg/mL	4.0 ± 0.084% *	1.10 ± 0.085%	10.65 ± 0.075%	84.25 ± 0.5%	11.75 ± 0.40%

## Abbreviations

CRC	colorectal cancer
DCFH-DA	2′,7′-dichlorofluorescein diacetate
DMSO	dimethyl sulfoxide
HO-1	Heme oxygenase-1
LDH	Lactic dehydrogenase
ROS	Reactive oxygen species
LOOH	Lipid hydroperoxide
RSH	Thiol groups
γ-GCS	γ-glutamylcysteine synthetase
